# Delegates to the National Medical Convention of 1847

**Published:** 1846-10

**Authors:** 


*Delegates to the National Medical Convention of* 1847.—We per-cerceive that Medical Societies in several States have already elec-ted delegates to the great convention to be holden in Philadelphia, in May next. We have understood that it is a subject of regret with many, that formal invitation was not extended to members of the profession generally throughout the country, who may be inclined to attend the convention, to participate in its deliberations. If we may judge from the spirit manifested at the last convention, there will be no disposition to be exclusive in any sense, and we presume medical men of respectability, who may be present, whether regularly appointed delegates or not, will not only have seats accorded to them, but the liberty of discussion if they desire it.

